# Supplementary material for: Ergosta-7,9(11),22-trien-3β-ol Alleviates Intracerebral Hemorrhage-Induced Brain Injury and BV-2 Microglial Activation
Source: Molecules. 2021 May 17;26(10):2970. doi: 10.3390/molecules26102970 (PMC8156058; doi:10.3390/molecules26102970)
Supplement: Supplementary file 1 [file molecules-26-02970-s001.zip › molecules-1188504-supplementary.pdf]

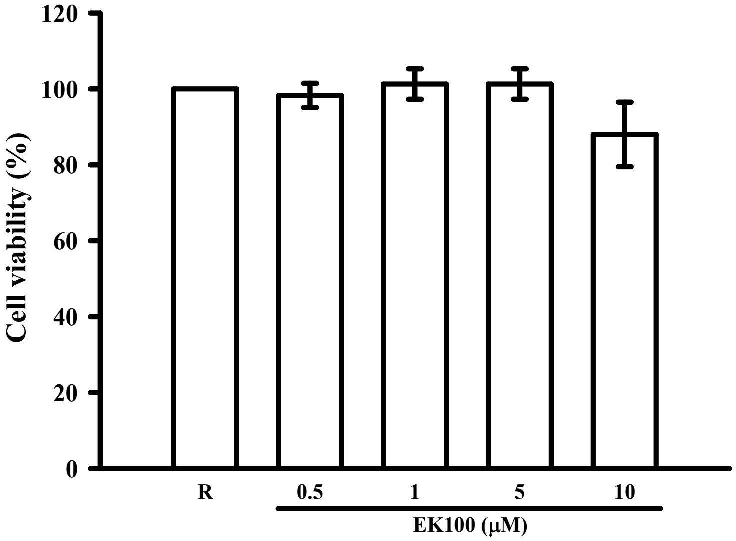

Supplementary Figure S1. Effects of EK100 on BV-2 microglial viability. Cellular viability was measured using a spectrophotometric assay at 550 nm, which was based on the ability of mitochondria to reduce the MTT in the viable cells. The percentage of BV-2 cell viability was calculated and presented as the mean±SD (n=3).

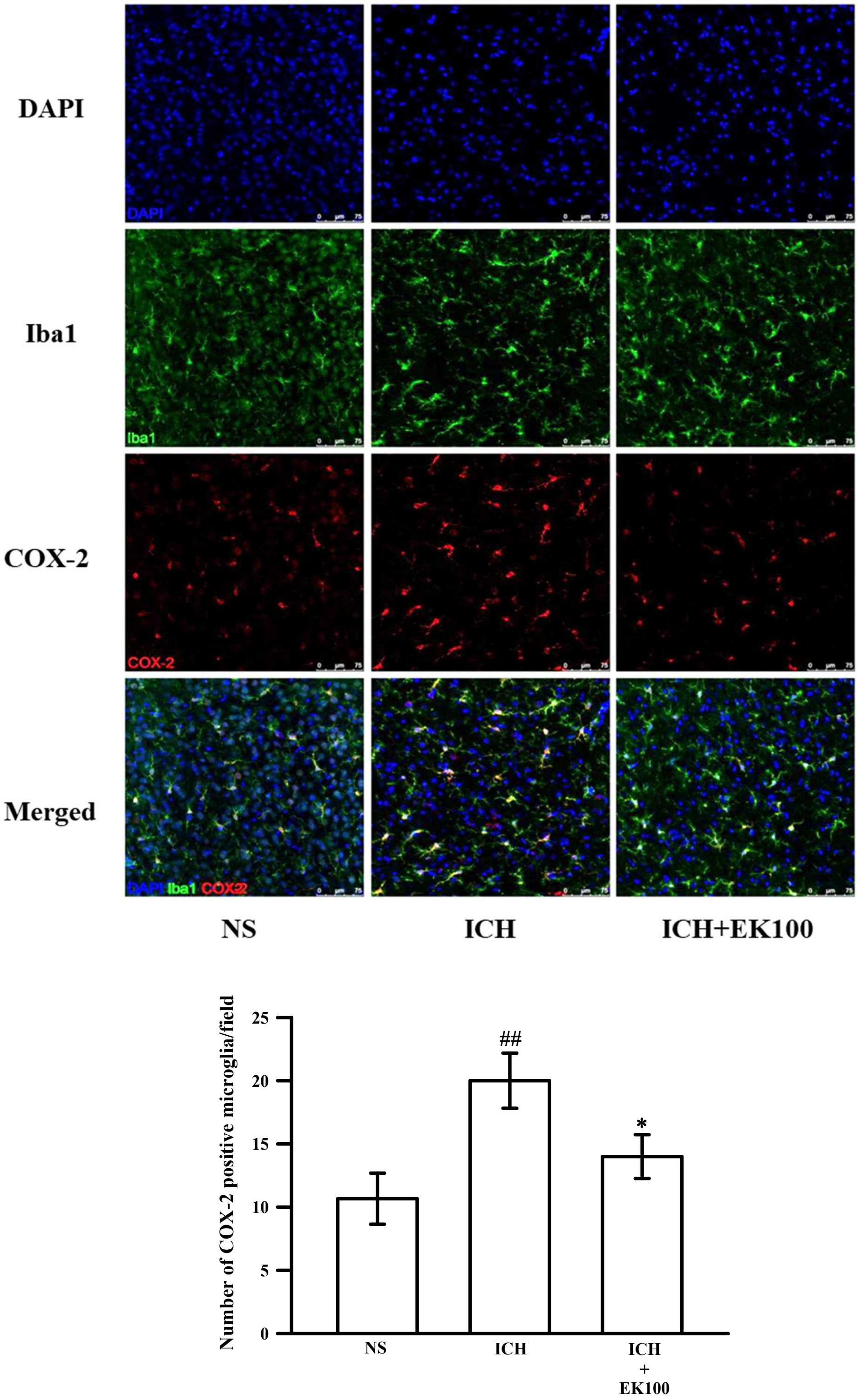

Supplementary Figure S2. Effects of EK100 on microglial COX-2 expression in the brain lesions after ICH. C57BL/6 mice were post-ICH orally administered EK100 (30 mg/kg) or vehicle at 30 minutes after ICH. After 24 h of ICH, manipulated brain sections (50 μm) were obtained for immunostaining studies. The brain sections were evaluated by a Leica TCS SP5 confocal microscope imaging system (Leica

Microsystems, Wetzlar, Germany). The cerebral cells (DAPI-positive cells), microglia (Iba1-positive cells) and COX-2-positive cells around the injured sites were evaluated (n=3). Positive (triple immunoreactive) cells were counted from two indicated areas (ipsilateral upper and tail corpus callosum) per animals. The quantitative analyses of the numbers of Iba-1 and COX-2 colocalization were performed by ImageJ software. The numbers were averaged and expressed as positive cells per field. Values were represented as the mean ± SD (n=3). $^{\#\#}p < 0.01$ compared with ipsilateral NS-intrastriatal injected with vehicle treatment group; $^{*}p < 0.05$ compared with ipsilateral collagenase-intrastriatal injected with vehicle treatment group. Bar indicates 75 μm.
